# Editorial: Global green strategies and capacities to manage a sustainable animal biodiversity

**DOI:** 10.3389/fgene.2023.1213080

**Published:** 2023-06-16

**Authors:** F. Perini, S. Ceccobelli, R. P. M. A. Crooijmans, C. K. Tiambo, E. Lasagna

**Affiliations:** ^1^ Department of Agricultural, Food and Environmental Sciences, University of Perugia, Perugia, Italy; ^2^Department of Agricultural, Food and Environmental Sciences, Università Politecnica Delle Marche, Ancona, Italy; ^3^ Animal Breeding and Genomics, Wageningen University and Research, Wageningen, Netherlands; ^4^ Centre for Tropical Livestock Genetics and Health, International Livestock Research Institute, Nairobi, Kenya

**Keywords:** animal genetic resources, conservation, sustainability, local breeds, molecular markers

Livestock animals have been domesticated and raised by humans for thousands of years, providing food, fiber, and other resources (Bruford et al., 2003). Over time, farmers have selectively bred animals to improve their productive traits (Rauw et al., 1998). However, the artificial selection process has also contributed significantly to the loss of genetic diversity (Ajmone-Marsan et al., 2023). Therefore, the current Research Topic was planned and researchers around the globe were invited to explore strategies to sustainably manage animal biodiversity. This Research Topic collected a total of eight manuscripts, published by 60 authors, more than 3,331 downloads and 14 thousand views globally at the time of this editorial. Keeping in view the importance of genetic diversity for a long-term potential for survival in different livestock species, we received manuscripts regarding cattle, sheep, chickens, and camels.

Among cattle, dairy animals are particularly prone to environmental stressors because they have been exposed to intense artificial selection for milk production and composition traits, thus losing the genetic variability that could be useful for new breeding orientations in the context of climate change (Leroy, 2014; Doublet et al., 2019). Indeed, Cheruiyot et al. proposed to include in the commercial cattle SNPs chip arrays some variants associated with the nervous system and metabolic functions that are responsible for heat tolerance in dairy cattle. Dairy cattle are also the most criticized for having a rather high environmental impact associated with greenhouse gases emissions such as nitrous oxide and methane (Webster, 2021). Genetic selection is a very attractive solution to identify and breed lower-methane emitter animals, however, it requires multidisciplinary studies and a large number of methane records from individual animal. Various studies have identified genetic markers linked with methane emissions in cattle and sheep, providing opportunities for selective breeding to develop animals with reduced methane production potential (Calderón-Chagoya et al., 2019; Zhang et al., 2020; Hickey et al., 2022). On the other hand, the urinary nitrogen release leads to high levels of environmental pollution (Uwizeye et al., 2020). Accordingly, Honerlagen et al. have studied, through a GWAS approach, the genomic features behind this phenotype, and found genomic regions and candidate genes affecting nitrogen excretion that could be included in a breeding selection scheme for animals with lower environmental impact. For a faster progress, genome editing techniques can improve livestock production efficiency and reduce environmental impacts that could have otherwise excessive cost using conventional breeding; however ethical issues regarding animal genome editing are still under debate and need to be concluded (Hallerman et al., 2022).

Specialized breeds are more likely to suffer from homozygosity, which can have a negative impact on genetic variability (Muir et al., 2008; Bosse et al., 2019). This loss of genetic diversity can lead to reduced adaptability, increased vulnerability to disease, and decreased resilience to environmental stressors (Grandin and Deesing, 2022). The conservation of genetic biodiversity in livestock animals has become a priority for a long-term sustainable agriculture (Pauls et al., 2013; DeLonge et al., 2016). Biodiversity is also crucial for ensuring adaptability to environmental stressors (Perini et al., 2020; Rovelli et al., 2020). Genomic research has provided the tools for deep characterization of genes responsible for certain phenotypes. Starting from the microsatellites, many QTLs related to interesting traits were mapped on chromosomes of livestock species (Hu et al., 2013; Badbarin et al., 2021). Moreover, the microsatellite technology, as well as the mitochondrial DNA, have been widely used to rapidly evaluate the genetic variability among and within breeds belonging to the same species (Perini et al., 2023a), and for the evaluation of their evolutionary relationships (Lasagna et al., 2020). The evolution of technology has led to the era of SNPs, a genomic tool much sharper for phenotype-genotype linkage identification. This is the case of Ben-Jemaa et al. that provides a glimpse into diverse selection signatures in semi-feral Maremmana cattle breed. The results showed a set of genes that are associated with the Maremmana breed’s ability to adapt to the environment of the western-central part of Italy.

Camels are a species that used to cope with semi-arid and arid environments and their characterization regarding phenotype, microsatellites, mitochondrial DNA and SNPs have been reviewed by Yakubu et al. The authors focused on the difficulties into distinguishing sub-populations in many countries, except for the populations in Kenya. Yakubu et al. also reported that several studies have been conducted mainly to investigate the association between SNPs markers and different traits.

Since SNPs technology is used as marker of genetic variability within and between breeds (Ceccobelli et al., 2023), the study by Van Marle-Köster et al. investigated inbreeding levels and the genetic architecture of South African cattle and sheep breeds. The authors found an abundance of short ROH fragments in both species indicating ancient inbreeding. Using principal component analysis, model-based clustering, and phylogenetic analyses, the eight cattle populations studied were classified into indicine, taurine, or Sanga subspecies. Considering the sheep population, a distinct separation among dual-purpose, meat, and indigenous breeds was observed.

Since the percentage of livestock breeds at risk of extinction has increased from 15% to 17% between 2005 and 2014 (FAO, 2015), genomic characterization of indigenous breeds is an important start of point for their conservation. Soglia et al. examined the genetic diversity and the rate of extinction of 17 native Italian chicken breeds starting from microsatellite genotyping. The study revealed that 11 breeds were in endangered status. These findings highlighted the need to preserve unique genetic diversity and special attention should be given to the introduction of different genetic lines and the use of mating schemes as a conservation strategy aimed at limiting the inbreeding increase.

RNA-sequencing is a tool which sheds light on global gene expressions of a given tissue, and it gave the possibility to discover new genomic variants with high impact on specific traits. It is also used to underline the adaptation of local livestock breeds to different environmental challenges (Perini et al., 2023b). RNA-seq has been used by Michailidou et al. for the evaluation of mammary gland transcriptional activity comparing high and low yielding ewes of two dairy sheep breeds reared intensively in Greece, the indigenous Chios, and the cosmopolitan Lacaune breed. The study demonstrated that the innate transcriptional activity of the mammary gland and the health status set the base for ewe productivity.

Finally, Peixoto et al. focused on the improvement of productive traits in Guzerá cattle. Due to its adaptation to harsh environments and low feed quality, parasite resistance, and dual-purpose characteristics, in 1994 breeding programs were implemented for the improvement of Guzerá for dairy purposes, using two joint selection strategies, the progeny test and the MOET nucleus schemes. The study provided evidence of the significant contribution of the MOET nucleus scheme to the breed’s phenotypic and genetic progress for milk traits.

This Research Topic has achieved its goal of kick-starting the discussion and utilization the resources in terms of which strategies and capacities will be more efficient to solve the primary challenges that are affecting animal biodiversity around the world.

## References

[B1] Ajmone-Marsan P. Boettcher P. J. Ginja C. Kantanen J. Lenstra J. A. (2023). Genomic characterization of animal genetic resources. FAO Animal Prod. Health Guidel No. 32. Rome. 10.4060/cc3079en

[B2] Badbarin S. Mirhoseini S. Z. Rabiei B. Hossein-Zadeh N. G. Khamisabadi H. Asroosh F. (2021). QTLs detection for mohair traits in Iranian Angora goats (Markhoz goats). Small Rumin. Res. 202, 106460. 10.1016/j.smallrumres.2021.106460

[B3] Bosse M. Megens H. J. Derks M. F. de Cara Á. M. Groenen M. A. (2019). Deleterious alleles in the context of domestication, inbreeding, and selection. Evol. Appl. 12, 6–17. 10.1111/eva.12691 30622631 PMC6304688

[B4] Bruford M. W. Bradley D. G. Luikart G. (2003). DNA markers reveal the complexity of livestock domestication. Nat. Rev. Genet. 4, 900–910. 10.1038/nrg1203 14634637

[B5] Calderón-Chagoya R. Hernandez-Medrano J. H. Ruiz-López F. J. Garcia-Ruiz A. Vega-Murillo V. E. Montano-Bermudez M. (2019). Genome-wide association studies for methane production in dairy cattle. Genes 10 (12), 995. 10.3390/life12071070 31810242 PMC6969927

[B6] Ceccobelli S. Landi V. Senczuk G. Mastrangelo S. Sardina M. T. Ben-Jemaa S. (2023). A comprehensive analysis of the genetic diversity and environmental adaptability in worldwide Merino and Merino-derived sheep breeds. Genet. Sel. Evol. 55, 24. 10.1186/s12711-023-00797-z 37013467 PMC10069132

[B7] DeLonge M. S. Miles A. Carlisle L. (2016). Investing in the transition to sustainable agriculture. Environ. Sci. Policy 55, 266–273. 10.1016/j.envsci.2015.09.013

[B8] Doublet A. C. Croiseau P. Fritz S. Michenet A. Hozé C. Danchin-Burge C. (2019). The impact of genomic selection on genetic diversity and genetic gain in three French dairy cattle breeds. Genet. Sel. Evol. 51, 52. 10.1186/s12711-019-0495-1 31547802 PMC6757367

[B22] FAO (2015). in The second report on the state of the world’s animal genetic resources for food and agriculture. Editors Scherf B. D. Pilling D. FAO Commission on Genetic Resources for Food and Agriculture Assessments. Rome. Available online at http://www.fao.org/3/a-i4787e/index.html .

[B9] Grandin T. Deesing M. J. (2022). “Genetics and animal welfare,” in Genetics and the behavior of domestic animals (Amsterdam, NL: Elsevier Press), 507–548. 10.1016/B978-0-323-85752-9.00013-5

[B10] Hallerman E. M. Bredlau J. P. Camargo L. S. A. Dagli M. L. Z. Karembu M. Ngure G. (2022). Towards progressive regulatory approaches for agricultural applications of animal biotechnology. Transgenic Res. 31, 167–199. 10.1007/s11248-021-00294-3 35000100 PMC8742713

[B11] Hickey S. M. Bain W. E. Bilton T. P. Greer G. J. Elmes S. Bryson B. (2022). Impact of breeding for reduced methane emissions in New Zealand sheep on maternal and health traits. Front. Genet. 13, 910413. 10.3389/fgene.2022.910413 36246641 PMC9561099

[B12] Hu Z. L. Park C. A. Wu X. L. Reecy J. M. (2013). Animal QTLdb: An improved database tool for livestock animal QTL/association data dissemination in the post-genome era. Nucleic Acids Res. 41, D871–D879. 10.1093/nar/gks1150 23180796 PMC3531174

[B13] Lasagna E. Ceccobelli S. Cardinali I. Perini F. Bhadra U. Thangaraj K. (2020). Mitochondrial diversity of Yoruba and fulani chickens: A biodiversity reservoir in Nigeria. Poult. Sci. 99, 2852–2860. 10.1016/j.psj.2019.12.066 32475418 PMC7597645

[B14] Leroy G. (2014). Inbreeding depression in livestock species: Review and meta-analysis. Anim. Genet. 45, 618–628. 10.1111/age.12178 24975026

[B15] Muir W. M. Wong G. K. S. Zhang Y. Wang J. Groenen M. A. Crooijmans R. P. (2008). Genome-wide assessment of worldwide chicken SNP genetic diversity indicates significant absence of rare alleles in commercial breeds. Proc. Natl. Acad. Sci. 105, 17312–17317. 10.1073/pnas.0806569105 18981413 PMC2582254

[B16] Pauls S. U. Nowak C. Bálint M. Pfenninger M. (2013). The impact of global climate change on genetic diversity within populations and species. Mol. Ecol. 22, 925–946. 10.1111/mec.12152 23279006

[B17] Perini F. Cardinali I. Ceccobelli S. Gruppetta A. San José C. Cosenza M. (2023a). Phylogeographic and population genetic structure of hound-like native dogs of the Mediterranean Basin. Res. Vet. Sci. 155, 103–114. 10.1016/j.rvsc.2023.01.010 36669378

[B18] Perini F. Cendron F. Rovelli G. Castellini C. Cassandro M. Lasagna E. (2020). Emerging genetic tools to investigate molecular pathways related to heat stress in chickens: A review. Animals 11, 46. 10.3390/ani11010046 33383690 PMC7823582

[B19] Perini F. Wu Z. Cartoni Mancinelli A. Soglia D. Schiavone A. Mattioli S. (2023b). RNAseq reveals modulation of genes involved in fatty acid biosynthesis in chicken liver according to genetic background, sex, and diet. Anim. Genet. 00, 1–17. 10.1111/age.13299 36752047

[B20] Rauw W. M. Kanis E. Noordhuizen-Stassen E. N. Grommers F. J. (1998). Undesirable side effects of selection for high production efficiency in farm animals: A review. Livest. Prod. Sci. 56, 15–33. 10.1016/S0301-6226(98)00147-X

[B21] Rovelli G. Ceccobelli S. Perini F. Demir E. Mastrangelo S. Conte G. (2020). The genetics of phenotypic plasticity in livestock in the era of climate change: A review. Ital. J. Anim. Sci. 19, 997–1014. 10.1080/1828051X.2020.1809540

[B23] Uwizeye A. De Boer I. J. M. Opio C. I. Schulte R. P. O. Falcucci A. Tempio G. (2020). Nitrogen emissions along global livestock supply chains. Nat. Food 1, 437–446. 10.1038/s43016-020-0113-y

[B24] Webster J. (2021). Green milk from contented cows: Is it possible? Front. Anim. Sci. 2, 667196. 10.3389/fanim.2021.667196

[B25] Zhang Q. Difford G. Sahana G. Løvendahl P. Lassen J. Lund M. S. (2020). Bayesian modelling reveals host genetics associated with rumen microbiota jointly influence methane emission in dairy cows. ISME J. 14 (8), 2019–2033. 10.1038/s41396-020-0663-x 32366970 PMC7368015

